# Variations in Suicide Risk and Risk Factors After Hospitalization for Depression in Finland, 1996-2017

**DOI:** 10.1001/jamapsychiatry.2023.5512

**Published:** 2024-02-14

**Authors:** Kari Aaltonen, Reijo Sund, Christian Hakulinen, Sami Pirkola, Erkki Isometsä

**Affiliations:** 1 Department of Psychiatry, University of Helsinki and Helsinki University Central Hospital, Helsinki, Finland; 2 Institute of Clinical Medicine, University of Eastern Finland, Kuopio, Finland; 3 Department of Psychology and Logopedics, University of Helsinki, Helsinki, Finland; 4 Finnish Institute for Health and Welfare, Helsinki, Finland; 5 Faculty of Social Sciences, University of Tampere and Pirkanmaa Hospital District, Tampere, Finland

## Abstract

**Importance:**

Although incidence of suicide in depression varies remarkably temporally, risk factors have been modeled as constant and remain uncharted in the short term. How effectively factors measured at one point in time predict risk at different time points is unknown.

**Objective:**

To examine the absolute risk and risk factors for suicide in hospitalized patients with depression starting from the first days after discharge up to 2 years and to evaluate whether the size of relative risk by factor displays temporal patterns over consecutive phases of follow-up.

**Design, Setting, and Participants:**

This population-based study using Finnish registers (hospital discharge, population, and cause of death registers) included all hospitalizations for depression as the principal diagnosis in Finland from 1996 to 2017, with a maximum follow-up of 2 years. Data were analyzed from January 2022 to November 2023.

**Main Outcomes and Measures:**

Incidence rate (IR), IR ratios, hazard functions, and hazard ratios for suicide by consecutive time periods (0 to 3 days, 4 to 7 days, 7 to 30 days, 31 to 90 days, 91 to 365 days, and 1 to 2 years) since discharge.

**Results:**

This study included 193 197 hospitalizations among 91 161 individuals, of whom 51 197 (56.2%) were female, and the mean (SD) age was 44.0 (17.3) years. Altogether, patients were followed up to 226 615 person-years. A total of 1219 men and 757 women died of suicide. Incidence of suicide was extremely high during the first days after discharge (IR of 6062 [95% CI, 4963-7404] per 100 000 on days 0 to 3; IR of 3884 [95% CI, 3119-4835] per 100 000 on days 4 to 7) and declined thereafter. Several factors were associated with risk of suicide over the first days after discharge. Current suicide attempt by hanging or firearms increased the risk of suicide most on days 0 to 3 (IR ratio, 18.9; 95% CI, 3.1-59.8) and on days 0 to 7 (IR ratio, 10.1; 95% CI, 1.7-31.5). Temporal patterns of the size of the relative risk diverged over time, being constant, declining, or increasing. Clinical factors had the strongest association immediately. Relative risk remained constant among men and even increased among those with alcohol or substance use disorder.

**Conclusions and Relevance:**

In this study, patients hospitalized for depression had extremely high risk of suicide during the first days after discharge. Thereafter, incidence declined steeply but remained high. Within the periods of the highest risk of suicide, several factors increased overall risk manyfold. Risk factors’ observed potencies varied over time and had characteristic temporal patterns.

## Key Points

**Question:**

What are the immediate and long-term risks and risk factors for suicide after hospitalization for patients with depression, and are there factor-specific time-dependent risks?

**Findings:**

In this cohort study including all 193 197 hospitalizations for depression in Finland from 1996 to 2017, patients hospitalized for depression had extremely high risk of suicide in the first days after discharge, with 7 factors modifying overall risk manyfold. The relative risk of each factor showed diverging temporal patterns, being constant, declining, or increasing.

**Meaning:**

Discharged patients with depression form a very high-risk group for immediate suicide, with the risk influenced by several factors with characteristic temporal patterns of observed potency.

## Teaser

This cohort study examines the absolute risk and risk factors for suicide in hospitalized patients with depression starting from the first days after discharge up to 2 years and evaluates whether the size of relative risk by factor displays temporal patterns over consecutive phases of follow-up.

## Introduction

Of all factors, depression is associated with the highest risk of suicide.^1^ Of all people dying of suicide, more than half had depression,^2^ and up to 7% to 8% of men and 4% of women with severe depression die of suicide.^3,4^ Of all people dying of suicide, approximately 40% had been hospitalized.^5,6^ After discharge, the risk of suicide in the first week and up to a month is highest,^7,8^ thereafter gradually declining.^3,9^ Arguably, the risk after discharge is highest among those with depression relative to other disorders,^5,6,7,10^ although limited studies have investigated illness-specific rates.^9^ Such a population with a distinct high-risk period in contact with psychiatric care forms a prioritizable target for selective suicide prevention.

Studies of risk factors of suicide in the coming days or week have not been feasible, despite a potentially pivotal role in clinical decision-making and care planning. In more than half of all suicides, the first lifetime attempt is lethal.^11,12^ In more than 80% of suicides with previous attempts, the fatal attempt occurs within 1 year of the first.^11^ By contrast, only a minority of all who have survived a suicide attempt eventually die of suicide.^3,13^ Since surviving and dying by a suicide attempt represent partly overlapping populations with differences in characteristics, caution is needed when generalizing findings from proxy studies of those who attempt suicide to those who die of suicide.^13,14^ Risk factors for suicide may, to a varying degree, be specific to disorders, but most studies are not illness specific^14,15^ or the scarce depression-specific evidence is limited to long-term risk factors.^3,16^ A systematic review yielded few consistent findings on risk factors for suicide within a year of discharge from a psychiatric hospital.^8^ The low incidence of suicides even among these high-risk populations necessitates enormous samples in risk factor studies.

The success of research on risk factors for suicide over the past decades has been compromised by several methodological constraints.^15^ Key limitations include static modeling over long-term follow-ups and a lack of studies on imminent risk factors. Such approach contrast with current understanding of suicidal behavior influenced by volatile state-dependent factors, like depression and traitlike predisposing or indirect factors.^13,17,18,19,20^ In addition, social factors contribute to suicidal diathesis and selection of lethal method on outcome^17,21^ but are subject to change. Whether characteristic risk factors for imminent suicide exists and, if so, the extent to which they retain their role for an extended period are unknown. More than 3 decades ago, Fawcett et al^22^ investigated time-related predictors for suicide but were constrained by a low base rate. Temporal variations have been investigated via timing of suicidal ideation by ecological momentary assessment^23^ and via suicide attempts by life-chart methodology^13^ instead of evaluating actual suicides. Neglecting time-dependent risks when summarizing varying lengths of follow-up data can potentially result in biased findings. The effects of clinical factors could be strongest in the short term since clinical states are variable and current states likely predict more accurately temporally close than distant states. Over time, the effects of factors could display diverging temporal patterns, influenced by the inherent tendency of a factor to change or by possible indirect associations. Neither imminent risk factors nor temporal patterns in any factor have been demonstrated to be linked to suicide deaths.

In this Finnish register-based study of patients hospitalized for depression from 1996 to 2017, we examined incidence of suicide starting from day 1 after discharge up to 2 years, risk factors for imminent suicide beginning over the first days after discharge, and variations in the size of a relative risk of a factor for risk of suicide during consecutive phases of follow-up.

## Methods

This Finnish register-based study identified all psychiatric hospitalizations of patients 18 years and older for depression from 1996 to 2017, and followed individuals up to 2 years from the last discharge. Individuals may have had several hospitalizations, and each discharge marked the beginning of a new follow-up period. The Ethics Committee of the Finnish Institute for Health and Welfare approved the study. Population registers were linked by personal identity codes at the individual level, with the permission of Statistics Finland and the Finnish Institute of Health and Welfare. For register-based studies, no informed consent is required. This study followed the Strengthening the Reporting of Observational Studies in Epidemiology (STROBE) reporting guideline.

### Care Register for Health Care

Episodes with depressive disorder as the principal diagnosis were identified from the Care Register for Health Care (CRHC) by the *International Statistical Classification of Diseases and Related Health Problems, Tenth Revision* (*ICD*-*10*) codes F32-33. Same-day discharges and readmissions were combined into 1 episode (the most serious diagnosis code defined the whole episode). We excluded patients with a comorbid diagnosis of a major psychotic disorder (*ICD*-*10* codes F20-29) or bipolar disorder (*ICD*-*10* code F31). Comorbid diagnoses of alcohol harmful use (*ICD*-*10* code F10.1) and alcohol dependence syndrome (*ICD*-*10* code F10.2; henceforth, alcohol use disorders [AUDs]) or of other substance harmful use (*ICD*-*10* codes F11.1 to F16.1, F18.1, and F19.1) and substance dependence syndrome (*ICD*-*10* codes F11.2 to F16.2, F18.2, and F19.2; henceforth, substance use disorders [SUDs]) were identified. Since 1996, national guidelines have instructed use of operationalized *ICD*-*10* Diagnostic Criteria for Research for clinical diagnoses, displaying good accuracy in the CRHC.^24^ For each index episode, we retrieved date of admission and discharge, overall severity of symptoms and functioning at admission by Global Assessment Scale (GAS) score, and whether the patient was involuntarily referred.

Nonfatal suicide attempts by each index episode were identified from the CRHC by *ICD*-*10* codes X60-X84, Y87.0, and Z91.5. Two distinct categories were modeled: (1) a current suicide attempt (requiring somatic admission immediately before or during the index episode or recorded in the psychiatric discharge notification) and (2) a previous suicide attempt within 4 years (from 1996 at the earliest). Data on suicide attempts include all hospital-treated attempts and from outpatient care since 1998.

### Population Data

Population data of Statistics Finland are annually updated. Individual-level sociodemographic data for each index hospitalization were retrieved on living alone (yes or no), household disposable income, and education level.

### Causes of Death

Statistics Finland maintains a register on dates and causes of death. Suicides were identified by *ICD*-*10* codes X60-X84, Y87.0, and Z91.5. The Finnish medicolegal death investigation standards yield reliable identification of suicides, leaving only a few undetermined deaths.^25^

### Follow-Up

Discharged patients were followed up on the registers for up to 2 years or until death, readmission, emigration, or December 2017, whichever came first. A readmission after 1 whole day of discharge denoted a new index episode. Other events than suicide, including diagnostic conversion to primary psychotic or bipolar disorder in the CRHC, were treated as censored.

The cause of death register includes data on place of death and the CRHC on deaths occurring during the hospital episode. Based on these data, we excluded those dying during hospitalization while retaining those dying subsequently on the day of discharge.

### Statistical Analysis

Hazard functions and hazard ratios for categorical covariates were estimated for the first 2 years by using a method based on penalized cubic splines.^26^ For continuous covariates, a 2-dimensional tensor was used to visualize the surface between follow-up time and covariate values. Suicide incidence was estimated with Poisson regression by splitting follow-up time to 3 days, 1 week, 1 month, 3 months, 1 year, and 2 years. Incidence rate ratios (IRRs) by covariate and multivariable Poisson models were estimated for both consecutive and cumulative time spans correspondingly. Cumulative incidence of suicide was estimated using the Aalen-Johansen estimator while considering other events as competing risks. Analyses were conducted using SAS version 9.4 (SAS Institute) and R version 4.2.2 (The R Foundation).

## Results

This study included 193 197 hospitalizations among 91 161 individuals, of whom 51 197 (56.2%) were female, and the mean (SD) age was 44.0 (17.3) years. The median (IQR) number of episodes per individual was 1 (1-2), and patients were followed up for a total of 226 615 person-years. The characteristics of index hospitalizations are presented in Table 1. There were a total of 1976 suicides.

**Table 1.** Characteristics of Index Episodes by Severity of Episode of Depression

Characteristic	No. (%)			
	Moderate depression^a^	Severe depression	Psychotic depression	Total population
Total hospitalizations	95 068 (49.2)	66 201 (34.3)	31 928 (16.5)	193 197 (100)
Sex				
Female	53 069 (55.8)	41 340 (62.4)	20 324 (63.7)	114 733 (59.4)
Male	41 999 (44.2)	24 861 (37.6)	11 604 (36.3)	78 464 (40.6)
AUD	20 900 (22.0)	7506 (11.3)	1514 (4.7)	29 920 (15.5)
SUD^b^	6186 (6.5)	1827 (2.8)	578 (1.8)	8591 (4.4)
GAS score				
50-100	31 579 (33.2)	15 796 (23.9)	5454 (17.1)	52 829 (27.3)
20-49	62 333 (65.6)	49 424 (74.7)	24 962 (78.2)	136 719 (70.8)
0-19	1156 (1.2)	981 (1.5)	1512 (4.7)	3649 (1.9)
Involuntary admission	8394 (8.8)	4470 (6.8)	7083 (22.2)	19 947 (10.3)
Suicide attempt^c^				
At index episode	4770 (5.0)	4177 (6.3)	1796 (5.6)	10 743 (5.6)
Hanging/firearm^d^	107 (0.1)	90 (0.1)	49 (0.2)	246 (0.1)
Poison/cutting^d^	4322 (4.5)	3859 (5.8)	1613 (5.1)	9794 (5.1)
Other^d^	341 (0.4)	228 (0.3)	134 (0.4)	703 (0.4)
In previous 4 y	12 880 (13.5)	8946 (13.5)	3496 (10.9)	25 322 (13.1)
Age, y				
18-39	42 386 (44.6)	24 923 (37.6)	9665 (30.3)	76 974 (39.8)
40-65	40 362 (42.5)	31 946 (48.3)	14 163 (44.4)	86 471 (44.8)
>65	12 320 (13.0)	9332 (14.1)	8100 (25.4)	29 752 (15.4)
Living alone	42 176 (44.4)	26 831 (40.5)	11 793 (36.9)	80 800 (41.8)
Household disposable income^e^				
Lowest tertile	34 910 (36.7)	19 088 (28.8)	9913 (31.0)	63 911 (33.1)
Middle tertile	52 774 (55.5)	40 345 (60.9)	18 949 (59.3)	112 068 (58.0)
Highest tertile	7384 (7.8)	6768 (10.2)	3066 (9.6)	17 218 (8.9)
Education				
Basic	38 793 (40.8)	22 717 (34.3)	13 086 (41.0)	74 596 (38.6)
Upper secondary	41 239 (43.4)	29 061 (43.9)	12 233 (38.3)	82 533 (42.7)
Tertiary^f^	15 036 (15.8)	14 423 (21.8)	6609 (20.7)	36 068 (18.7)
Duration of hospitalization, wk				
<1	36 631 (38.5)	16 331 (24.7)	5641 (17.7)	58 603 (30.3)
1-3	35 070 (36.9)	21 626 (32.7)	6927 (21.7)	63 623 (32.9)
>3	23 367 (24.6)	28 244 (42.7)	19 360 (60.6)	70 971 (36.7)
Suicides during follow-up, No.	917	710	349	1976

Abbreviations: AUD, alcohol use disorder; GAS: Global Assessment Scale; SUD, substance use disorder.

^a^ Includes all other types of depression (from mild to moderate, partial remissions, other specified, and unspecified).

^b^ Excluding alcohol or nicotine harmful use or dependence syndrome.

^c^ Categories not overlapping; an individual may have both.

^d^ By method of index suicide attempt.

^e^ Thresholds correspond to the levels of household disposable income of the entire population.

^f^ Includes lowest-level tertiary education or higher.

### Incidence

**Figure 1.**
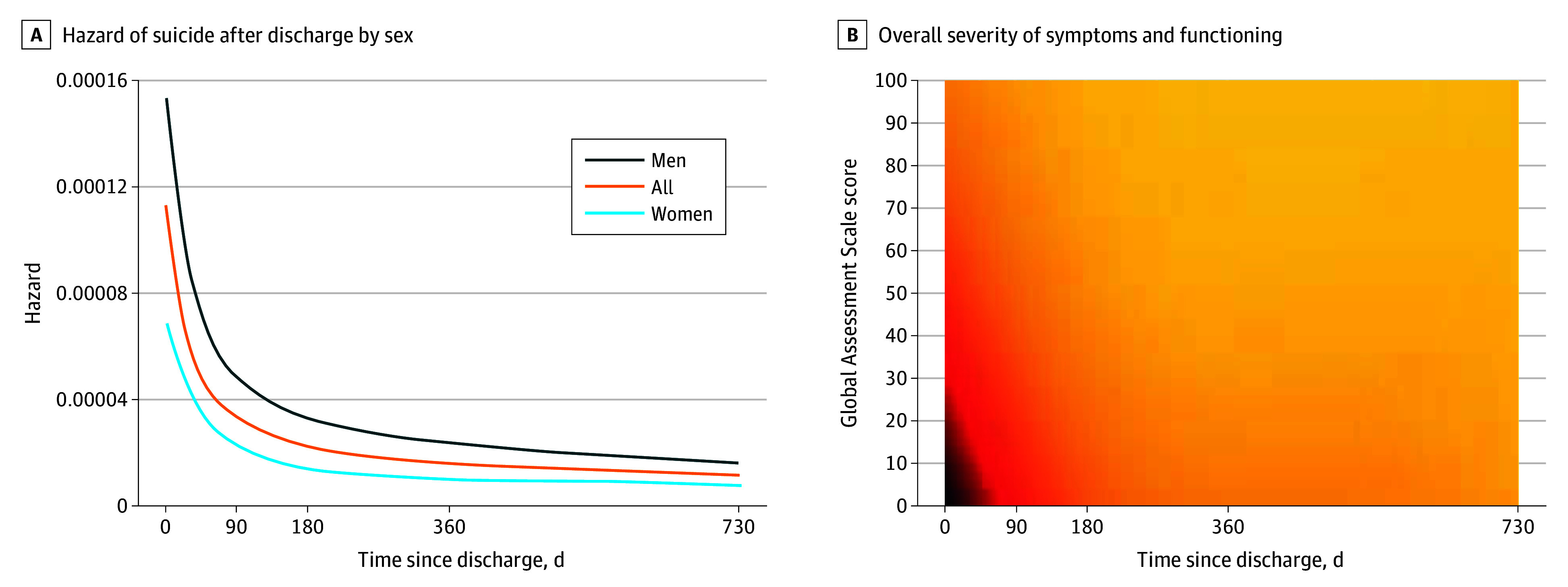
Hazard of Suicide After Discharge by Sex and Overall Severity of Symptoms and Functioning Up to 2 Years A, Hazard of suicide for men, women, and overall by time from discharge in days. B, The heat map represents risk of suicide by Global Assessment Scale score assigned at admission and time from discharge, where black indicates higher and yellow indicates lower relative risk.

The incidence of suicide is presented in Table 2, and the cumulative hazard of suicide is presented in Figure 1A. Incidence of suicide was extremely high during the first days after discharge (incidence rate [IR] of 6062 [95% CI, 4963-7404] per 100 000 on days 0 to 3; IR of 3884 [95% CI, 3119-4835] per 100 000 on days 4 to 7), with a marked sex difference (male: IR of 8551 [95% CI, 6565-11 138] per 100 000 on days 0 to 3; IR of 5743 [95% CI, 4328-7621] per 100 000 on days 4 to 7; female: IR of 4359 [95% CI, 3210-5920] per 100 000 on days 0 to 3; IR of 2614 [95% CI, 1849-3696] per 100 000 on days 4 to 7), thereafter declining. Excluding discharge day suicides, the IR was 5432 (95% CI, 3721-5869) per 100 000 on days 0 to 3. Of patients dying of suicide within 2 years, 176 of 1976 (8.9%) died during the first week and 448 (22.7%) during the first month.

**Table 2.** Incidence of Suicide by Time From Last Discharge

Time from discharge	Events, No.	Person-years at risk	Incidence rate per 100 000 person-years (95% CI)
All			
0-3 d	96	1583.7	6062 (4963-7404)
4-7 d	80	2059.9	3884 (3119-4835)
8-30 d	272	10 993.7	2474 (2197-2786)
31-90 d	381	24 663.8	1545 (1397-1708)
91-365 d	682	90 001.1	758 (703-817)
1-2 y	465	97 313.0	478 (436-523)
Men			
0-3 d	55	643.2	8551 (6565-11 138)
4-7 d	48	835.8	5743 (4328-7621)
8-30 d	165	4487.5	3677 (3157-4283)
31-90 d	232	10 288.9	2255 (1983-2565)
91-365 d	431	38 122.9	1131 (1029-1242)
1-2 y	288	41 517.6	694 (618-779)
Women			
0-3 d	41	940.6	4359 (3210-5920)
4-7 d	32	1224.2	2614 (1849-3696)
8-30 d	107	6506.2	1645 (1361-1988)
31-90 d	149	14 374.9	1037 (883-1217)
91-365 d	251	51 878.2	484 (428-548)
1-2 y	177	55 795.5	317 (274-368)

### Short-Term Risk Factors

The IRRs for suicide over consecutive periods after discharge by 12 different factors are presented in Table 3. Death from suicide within 3 days of discharge was associated with episodes of severe depression (IRR, 2.18; 95% CI, 1.37-3.52) and psychotic depression (IRR, 2.36; 95% CI, 1.35-4.07), higher severity of symptoms and impairment (GAS score of 0-19: IRR, 5.26; 95% CI, 1.46-15.39; GAS score of 20-49; IRR, 2.85; 95% CI, 1.59-5.66), index episode suicide attempt (IRR, 3.66; 95% CI, 2.09-6.02), earlier suicide attempt (IRR, 2.09; 95% CI, 128-3.29), AUD (IRR, 0.43; 95% CI, 0.18-0.86), male sex (IRR, 1.96; 95% CI, 1.31-2.96), age (40 to 65 years: IRR, 2.76; 95% CI, 1.68-4.75; older than 65 years: IRR, 2.45; 95% CI, 1.28-4.68), household disposable income level within the highest tertile (IRR, 1.99; 95% CI, 1.04-3.67), and year of hospitalization.

**Table 3.** Incidence Rate Ratios (IRRs) and Adjusted IRRs (aIRRs) for Suicide by Consecutive Periods Since Discharge^a^

Group	0-3 d		4-7 d		7-30 d		31-90 d		91-365 d		1-2 y	
	IRR (95% CI)	aIRR (95% CI)	IRR (95% CI)	aIRR (95%CI)	IRR (95% CI)	aIRR (95% CI)	IRR (95% CI)	aIRR (95% CI)	IRR (95% CI)	aIRR (95% CI)	IRR (95% CI)	aIRR (95% CI)
Sex												
Female	1 [Reference]	1 [Reference]	1 [Reference]	1 [Reference]	1 [Reference]	1 [Reference]	1 [Reference]	1 [Reference]	1 [Reference]	1 [Reference]	1 [Reference]	1 [Reference]
Male	1.96 (1.31-2.96)	2.28 (1.51-3.45)	2.20 (1.41-3.47)	2.24 (1.42-3.59)	2.24 (1.76-2.86)	2.38 (1.86-3.07)	2.18 (1.77-2.68)	2.21 (1.79-2.74)	2.34 (2.00-2.73)	2.39 (2.03-2.80)	2.19 (1.82-2.64)	2.04 (1.68-2.47)
Depression severity												
Moderate^b^	1 [Reference]	1 [Reference]	1 [Reference]	1 [Reference]	1 [Reference]	1 [Reference]	1 [Reference]	1 [Reference]	1 [Reference]	1 [Reference]	1 [Reference]	1 [Reference]
Severe	2.18 (1.37-3.52)	1.91 (1.18-3.13)	1.28 (0.79-2.07)	1.32 (0.79-2.19)	1.44 (1.11-1.87)	1.53 (1.16-2.00)	1.04 (0.83-1.31)	1.12 (0.88-1.42)	1.25 (1.06-1.48)	1.40 (1.17-1.67)	1.03 (0.84-1.26)	1.16 (0.94-1.44)
Psychotic	2.36 (1.35-4.07)	1.87 (1.03-3.38)	1.20 (0.62-2.17)	1.22 (0.60-2.36)	1.17 (0.82-1.64)	1.13 (0.77-1.64)	1.25 (0.95-1.64)	1.42 (1.04-1.92)	1.37 (1.11-1.68)	1.44 (1.14-1.82)	1.05 (0.80-1.36)	1.19 (0.88-1.59)
AUD	0.43 (0.18-0.86)	0.32 (0.13-0.66)	1.68 (0.98-2.76)	1.25 (0.70-2.15)	1.11 (0.80-1.50)	0.79 (0.56-1.09)	1.59 (1.25-2.00)	1.14 (0.88-1.47)	1.47 (1.23-1.76)	1.07 (0.88-1.30)	1.58 (1.27-1.95)	1.11 (0.88-1.39)
SUD^c^	0.93 (0.29-2.23)	1.16 (0.35-2.83)	0.83 (0.20-2.22)	0.77 (0.19-2.10)	1.67 (1.02-2.56)	1.63 (0.99-2.54)	1.19 (0.74-1.80)	1.07 (0.66-1.64)	1.86 (1.41-2.42)	1.76 (1.31-2.30)	2.03 (1.45-2.76)	1.92 (1.36-2.65)
GAS score												
50-100	1 [Reference]	1 [Reference]	1 [Reference]	1 [Reference]	1 [Reference]	1 [Reference]	1 [Reference]	1 [Reference]	1 [Reference]	1 [Reference]	1 [Reference]	1 [Reference]
20-49	2.85 (1.59-5.66)	2.95 (1.61-5.93)	1.59 (0.93-2.90)	1.51 (0.87-2.80)	1.33 (1.00-1.80)	1.29 (0.96-1.77)	1.40 (1.09-1.81)	1.32 (1.02-1.72)	1.43 (1.18-1.74)	1.28 (1.05-1.57)	1.26 (1.01-1.59)	1.20 (0.96-1.52)
0-19	5.26 (1.46-15.39)	4.20 (1.12-12.98)	2.88 (0.67-8.73)	2.40 (0.54-7.69)	3.43 (1.84-5.98)	2.88 (1.50-5.19)	1.54 (0.72-2.91)	1.18 (0.54-2.28)	2.00 (1.22-3.09)	1.30 (0.79-2.05)	1.61 (0.86-2.76)	1.28 (0.68-2.25)
Suicide attempt^d^												
At index episode^e^	3.66 (2.09-6.02)	3.17 (1.77-5.37)	2.43 (1.18-4.50)	1.93 (0.92-3.69)	2.33 (1.59-3.31)	1.91 (1.29-2.75)	2.92 (2.19-3.83)	2.45 (1.82-3.26)	2.85 (2.30-3.50)	2.39 (1.91-2.96)	1.79 (1.31-2.40)	1.52 (1.10-2.05)
In past 4 y^e^	2.09 (1.28-3.29)	2.28 (1.36-3.70)	2.09 (1.21-3.42)	2.22 (1.26-3.76)	2.05 (1.53-2.70)	2.11 (1.55-2.83)	2.29 (1.80-2.89)	2.16 (1.67-2.76)	2.15 (1.78-2.58)	1.97 (1.62-2.39)	1.99 (1.56-2.50)	1.85 (1.44-2.36)
Involuntary admission	1.48 (0.81-2.53)	1.14 (0.60-2.00)	1.23 (0.59-2.24)	1.05 (0.49-2.00)	1.53 (1.09-2.11)	1.31 (0.91-1.83)	1.27 (0.94-1.69)	1.03 (0.75-1.38)	1.81 (1.49-2.18)	1.48 (1.21-1.80)	1.67 (1.32-2.09)	1.49 (1.16-1.89)
Age, y												
18-39	1 [Reference]	1 [Reference]	1 [Reference]	1 [Reference]	1 [Reference]	1 [Reference]	1 [Reference]	1 [Reference]	1 [Reference]	1 [Reference]	1 [Reference]	1 [Reference]
40-64	2.76 (1.68-4.75)	2.79 (1.67-4.88)	1.52 (0.94-2.52)	1.47 (0.89-2.49)	1.53 (1.17-2.00)	1.61 (1.22-2.13)	1.27 (1.03-1.58)	1.25 (1.00-1.58)	1.20 (1.02-1.41)	1.24 (1.05-1.47)	1.34 (1.11-1.63)	1.40 (1.14-1.71)
≥65	2.45 (1.28-4.68)	3.34 (1.68-6.63)	1.23 (0.60-2.40)	1.92 (0.90-3.91)	1.20 (0.81-1.74)	1.75 (1.16-2.60)	0.93 (0.66-1.29)	1.33 (0.93-1.89)	1.00 (0.78-1.27)	1.39 (1.07-1.80)	0.69 (0.47-0.96)	0.98 (0.66-1.40)
Living alone	0.80 (0.52-1.20)	0.87 (0.56-1.33)	0.63 (0.39-1.01)	0.55 (0.33-0.88)	0.83 (0.64-1.05)	0.73 (0.57-0.94)	1.03 (0.84-1.26)	0.93 (0.75-1.14)	1.25 (1.08-1.46)	1.09 (0.93-1.27)	1.32 (1.10-1.59)	1.21 (1.01-1.46)
Household disposable income level												
Lowest tertile	1 [Reference]	1 [Reference]	1 [Reference]	1 [Reference]	1 [Reference]	1 [Reference]	1 [Reference]	1 [Reference]	1 [Reference]	1 [Reference]	1 [Reference]	1 [Reference]
Middle tertile	1.08 (0.69-1.73)	0.83 (0.52-1.35)	0.56 (0.35-0.89)	0.44 (0.27-0.72)	0.68 (0.53-0.87)	0.56 (0.43-0.72)	0.68 (0.55-0.84)	0.61 (0.49-0.77)	0.60 (0.51-0.70)	0.56 (0.47-0.66)	0.75 (0.62-0.91)	0.76 (0.62-0.93)
Highest tertile	1.99 (1.04-3.67)	1.25 (0.62-2.46)	0.93 (0.42-1.85)	0.55 (0.24-1.18)	0.65 (0.39-1.02)	0.40 (0.23-0.64)	0.90 (0.62-1.26)	0.68 (0.46-0.99)	0.52 (0.37-0.70)	0.41 (0.29-0.56)	0.73 (0.51-1.02)	0.71 (0.49-1.02)
Education level												
Basic	1 [Reference]	1 [Reference]	1 [Reference]	1 [Reference]	1 [Reference]	1 [Reference]	1 [Reference]	1 [Reference]	1 [Reference]	1 [Reference]	1 [Reference]	1 [Reference]
Upper secondary	1.04 (0.65-1.65)	1.17 (0.72-1.89)	1.22 (0.74-2.05)	1.39 (0.82-2.37)	1.00 (0.76-1.32)	1.12 (0.85-1.49)	1.17 (0.93-1.47)	1.25 (0.99-1.59)	1.01 (0.85-1.19)	1.12 (0.94-1.34)	1.27 (1.04-1.56)	1.32 (1.07-1.62)
Tertiary^f^	1.40 (0.81-2.36)	1.29 (0.72-2.26)	1.52 (0.83-2.72)	1.90 (0.99-3.58)	1.40 (1.02-1.91)	1.82 (1.30-2.54)	1.41 (1.07-1.85)	1.71 (1.28-2.29)	1.19 (0.97-1.45)	1.65 (1.33-2.05)	1.01 (0.77-1.32)	1.18 (0.88-1.56)
Duration of hospitalization, wk												
<1	1 [Reference]	1 [Reference]	1 [Reference]	1 [Reference]	1 [Reference]	1 [Reference]	1 [Reference]	1 [Reference]	1 [Reference]	1 [Reference]	1 [Reference]	1 [Reference]
1-3	0.86 (0.52-1.43)	0.68 (0.41-1.14)	1.75 (1.00-3.16)	1.61 (0.92-2.92)	1.01 (0.75-1.36)	0.94 (0.70-1.28)	0.91 (0.71-1.18)	0.89 (0.69-1.15)	0.83 (0.69-1.01)	0.82 (0.67-1.00)	0.91 (0.72-1.14)	0.93 (0.73-1.17)
>3	0.96 (0.59-1.55)	0.56 (0.33-0.94)	1.21 (0.67-2.22)	1.05 (0.56-2.00)	0.90 (0.67-1.22)	0.78 (0.57-1.08)	0.86 (0.67-1.11)	0.82 (0.62-1.07)	0.97 (0.81-1.16)	0.91 (0.74-1.11)	0.84 (0.67-1.05)	0.87 (0.68-1.12)
Year of hospitalization												
1996-2002	1 [Reference]	1 [Reference]	1 [Reference]	1 [Reference]	1 [Reference]	1 [Reference]	1 [Reference]	1 [Reference]	1 [Reference]	1 [Reference]	1 [Reference]	1 [Reference]
2003-2009	0.62 (0.38-0.99)	0.57 (0.35-0.92)	0.75 (0.45-1.25)	0.71 (0.42-1.19)	0.69 (0.52-0.92)	0.64 (0.48-0.85)	0.85 (0.67-1.08)	0.79 (0.63-1.01)	0.84 (0.71-1.00)	0.76 (0.64-0.91)	0.87 (0.71-1.07)	0.81 (0.65-0.99)
2010-2017	0.56 (0.33-0.91)	0.46 (0.27-0.76)	0.64 (0.36-1.10)	0.58 (0.33-1.01)	0.66 (0.49-0.88)	0.57 (0.42-0.77)	0.71 (0.55-0.92)	0.63 (0.48-0.81)	0.66 (0.54-0.79)	0.56 (0.46-0.68)	0.65 (0.51-0.82)	0.58 (0.45-0.74)

Abbreviations: AUD, alcohol use disorder; GAS, Global Assessment Scale; SUD, substance use disorder.

^a^ Adjusted for all other variables.

^b^ Includes all other types of depression (from mild to moderate, partial remissions, other specified, and unspecified).

^c^ Excluding alcohol or nicotine use disorder or dependence.

^d^ Categories of suicide attempt at index episode and in past 4 years nonoverlapping; an individual may have both.

^e^ Any method.

### Temporal Variations in Relative Risks

The hazards of suicide after discharge are depicted for severity of symptoms and impairment (GAS score) in Figure 1B, for age in eFigure 1 in Supplement 1, and for other factors in eFigure 2 in Supplement 1.

**Figure 2.**
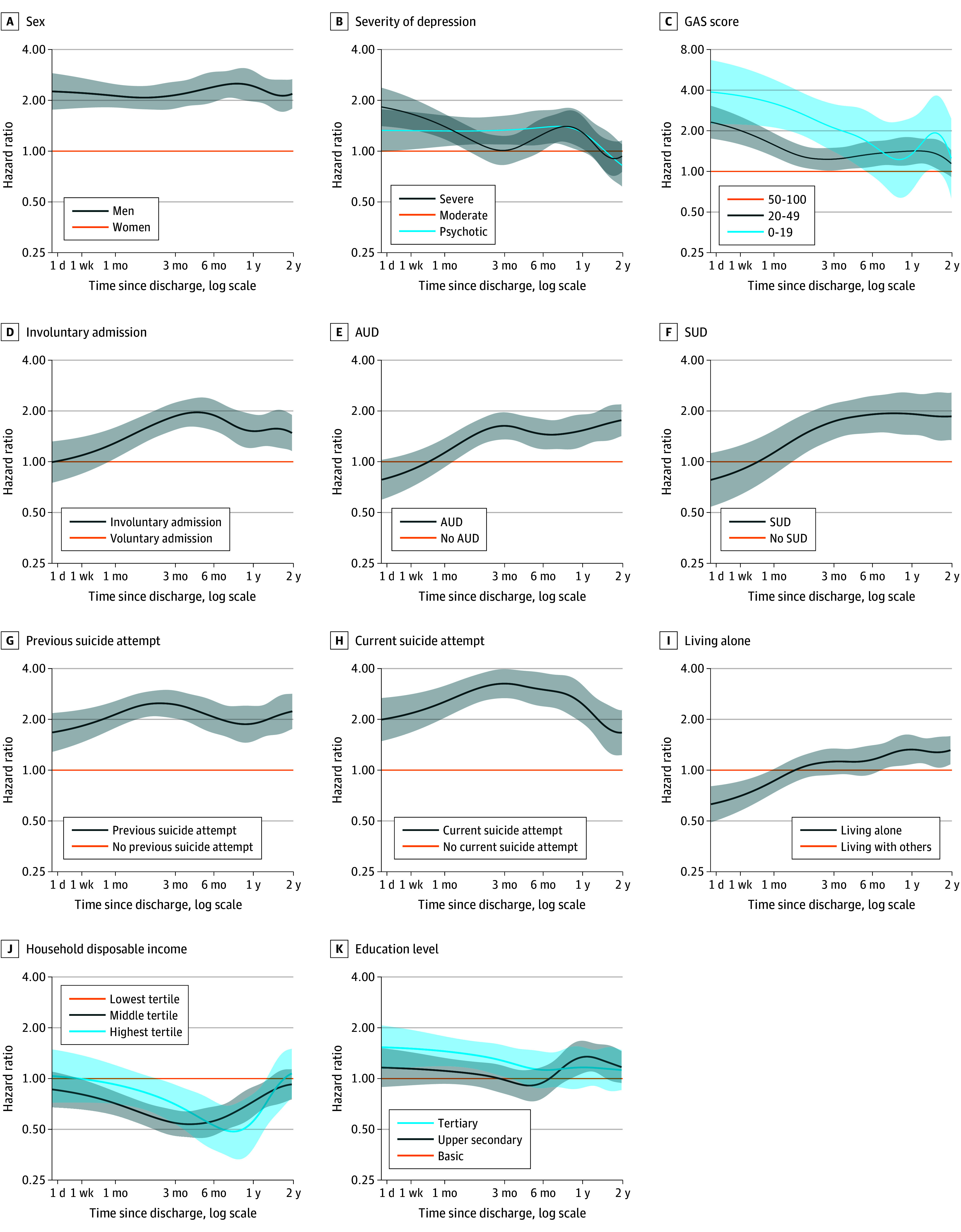
Variations in Relative Risk of Suicide by 11 Factors as a Continuum Up to 2 Years Curves represent instantaneous hazard ratios of suicide compared with the reference group, that is, reflect what hazard ratio is at each period after discharge (and how hazard ratios change in time). For example, panel A shows that risk of suicide is about 2-fold higher among men than among women throughout the first 2 years after discharge. Panel E shows that for people with alcohol use disorder (AUD), risk of suicide for the first month is about the same as with people without AUD but then rises in time and ends up being approximately 1.5-fold higher for later periods. GAS indicates Global Assessment Scale; SUD, substance use disorder.

The IRRs for suicide over consecutive subperiods of the 2-year follow-up by 12 factors are presented in Table 3 and by conventional cumulative time periods in the eTable in Supplement 1. The IRRs remained constant among men and patients with previous suicide attempts. The IRRs were initially high but later declined for severe or psychotic depression, current suicide attempt, lower GAS score, and age of 40 to 65 years or older than 65 years. AUD and living alone were associated with immediate suicide risk inversely but later directly. High household disposable income was associated with immediate suicide risk directly but later inversely. Involuntary admission was associated with higher risk only later. The temporal variations in hazard ratios by 11 factors are projected as a continuum over the follow-up in Figure 2.

## Discussion

This national longitudinal register-based study of all hospitalizations for depression in Finland over a 22-year period from 1996 to 2017 demonstrates the very high average risk of suicide pertaining to individuals hospitalized for depression. Postdischarge suicide risk was found to be the highest over the first days, then steeply declined. Even within this extremely high-risk period and high-risk population, several factors were associated with imminent suicide death, including plausible clinical factors, male sex, higher age, and higher household disposable income. Temporally, relative risk remained constant for men, decreased for clinical factors, and even increased for AUD and SUD. Hence, the absolute risk changed and the temporal patterns of observed relative risks also differed.

### Temporal Variations in Incidence of Suicide

The present findings are in line with a recent Danish national study^5^ reporting patients with depression have the highest suicide risk of all discharged patients. The Finnish incidence rates roughly compare with or exceed the corresponding Danish rates but markedly exceed the pooled risk of all discharged psychiatric patients.^7,9^ The incredibly high population risk could be explained by the critical role of depression, when those who are the most severely ill and often estimated to be at high risk of suicide become admitted.

The risk of suicide was most extreme immediately after discharge, when the 3-day and 7-day rates exceeded the mean general population rate in 1996 to 2017 by approximately 330-fold and 260-fold, respectively.^27^ Of those who had died on the date of discharge, previous studies have excluded all of them,^5,7^ whereas we excluded only those who died while still admitted. These 2 approaches may underestimate and overestimate, respectively, the true incidence.

Improved overall care for depression may have contributed to marked decline in long-term suicide mortality in patients hospitalized for severe depression.^28^ Although we found a decreasing trend over time, the high-risk postdischarge period still requires intensified attention. Contributing factors and targets for prevention could include a treatment failure, low response to treatments chosen, delays in response, or insufficiently identified risk during admission. Patients may conceal their intentions in the last appointments before death,^29^ and unplanned or patient-initiated discharges are risk factors for postdischarge suicide.^8,30^ Continuity of care and access to enhanced psychiatric outpatient care within days of discharge should be imperative.^31^

### Short-Term Risk Factors

Several risk factors for suicide within a few days were revealed by this longitudinal register-based study, of which findings complement those of psychological autopsy studies.^1^ The outcome was based on large-scale nationwide data, exceeding the numbers of suicides in pooled data.^8^

Clinical risk factors (severity of depressive episode, high illness severity and impairment, current suicide attempt) predominated the immediate risk together with age and male sex. Each factor indicated about 2-fold to 5-fold higher relative risk of suicide in the few days after discharge; since this occurred during a period of remarkably high overall incidence, this translated into exceedingly high absolute risk.

Previous findings on whether psychotic symptoms per se increase risk of suicide in those with severe depression have been mixed and dependent on study design.^32^ Among national studies, where individuals may belong to both groups,^33^ as here, no group differences stand out, whereas those with fixed classification after the first episode have reported added risk.^3,32,34,35^ Over the early but declining high-risk period, general assessment of symptom severity and functional impairment (GAS) was inversely associated with risk of suicide and time in the highest risk. Whether the role of illness severity in the short term has been underestimated due to crude, limited, and long follow-up data requires further clarification.

A strong gradient in imminent risk was found when suicide attempts at the index episode were grouped by estimated lethality of a given method,^21^ from intoxication or cutting to firearms or hanging, and other violent methods ranking in between these extremes. Alarmingly, aligning with other findings,^36^ in the minority of patients with a recent suicide attempt by hanging or firearm, the already very high population risk was multiplied 19-fold within 3 days and 10-fold within 7 days postdischarge.

Some short-term findings were counterintuitive. The inverse association with AUD or SUD is unexpected but consistent with recent findings in the medium term.^37^ Because individuals with AUD and SUD less often had severe depression, substance use may have contributed to the crisis leading to admission, which consequently served as detoxification. Confounding by illness severity or losses could explain the association between high income or living with others and imminent risk.^3,38,39^

### Temporal Patterns of Relative Risk

Men and those who had previously attempted suicide consistently had higher risk of suicide postdischarge. The former is plausible given the inherent nature of sex, presumably explained by more lethal methods chosen by men,^3,21^ whereas past suicide attempts could reflect preexisting diathesis for attempting suicide in case of recurrent depressive episodes.^40^

The high immediate but gradually decreasing relative risk as time passed pertained to acute clinical risk factors (severe or psychotic depression, severe illness with impaired function, current suicide attempt) and age. In 1 year, the relative risk of clinical factors had disappeared, and the risk associated with current suicide attempt had declined to a level comparable with having previously attempted suicide overall.

The pattern of increasing risk, showing either null or inverse associations at first but after 1 to 3 months entailing higher risk, pertained to involuntary admission, AUD, SUD, and living alone. AUD and SUD were associated with rising associations. At first, confounding by illness severity or treatment effects may have had an impact. In the longer term, involuntary admission could serve as a proxy for more complex disorders, impaired self-control, and suicidal diathesis. AUD, SUD, and loneliness are recognized long-term risk factors,^3,41^ with potential multifaceted and accumulative detrimental effects over time.

In the general population, low education and income are associated with higher risk of suicide.^42,43^ Opposite findings have been reported after discharge^3,38^ although these either did not apply to those hospitalized for depression^38^ or were based on a long-term follow-up.^3^ Here, low household income was clearly associated with higher overall risk; for education, no clear pattern emerged.

### Implications for Risk Factor Research

Shortcomings in accurate and timely measurement of risk factors have constrained study designs to rely on assessment at a given time and then modeling risk for varying lengths of follow-ups. Only clinical studies have prospectively modeled how changes in factors influence timing of nonfatal suicidal behavior.^13,23^ Unparalleled, we modeled time span for size of relative risk by each factor. The ability of any measure to predict suicide risk depends on both (1) true prospective variation of a factor, which almost always remains unknown, and (2) correlation of the baseline measure with future condition, which likely diminishes over time. We have demonstrated that the size of the relative risk by factor shows varying patterns over time but cannot differentiate between 2 intertwined, plausible reasons. Nevertheless, because some relative risks in fact increased rather than declined, this excludes the mere influence of increasing imprecision of prediction over time.

### Limitations

Limitations of this study are those common to register-based studies,^14^ including crudeness of available data and longitudinal course of depression remaining unknown. Diagnostic assessments characterize patients’ clinical status at their worst point, overlooking recoveries and clinical status at discharge. In clinical settings, psychotic depression, AUD, and SUD remain underdiagnosed. After involuntary admission, treatment may have continued voluntarily or involuntarily or with the patient being discharged after the observation period. Information on previous suicide attempts is not complete. Socioeconomic status was subject to change prior to admission or death. Confounding factors not captured by register data could exist.^1^ Most inaccuracies likely underestimate true prevalence or effect sizes. Some misclassifications may remain between those discharged and still admitted when the date of death corresponded to the last day of the episode.

## Conclusions

Discharged patients with depression form a very high-risk group for immediate suicide, the risk being the highest over the first postdischarge days. Several factors modify the very high short-term risk. Over time, factor-specific relative risks of suicide in depression show temporal variations, either constant, decreasing, or increasing. Future studies must address risk factors as temporally dynamic.

## Notes

Supplement 1. **eTable. **Unadjusted and Adjusted Incidence Rate Ratios for Suicide by Cumulative Time Since Discharge **eFigure 1. **Hazard of Suicide by Age and Days Since Discharge Up to 2 Years **eFigure 2. **Hazard of Suicide by Days Since Discharge By 11 Factors Up to 2 Years

Supplement 2. Data Sharing Statement

## References

[1] Favril L, Yu R, Uyar A, Sharpe M, Fazel S. Risk factors for suicide in adults: systematic review and meta-analysis of psychological autopsy studies. Evid Based Ment Health. 2022;25(4):148-155. doi:10.1136/ebmental-2022-300549 36162975 PMC9685708

[2] Cho SE, Na KS, Cho SJ, Im JS, Kang SG. Geographical and temporal variations in the prevalence of mental disorders in suicide: systematic review and meta-analysis. J Affect Disord. 2016;190:704-713. doi:10.1016/j.jad.2015.11.008 26600412

[3] Aaltonen KI, Isometsä E, Sund R, Pirkola S. Risk factors for suicide in depression in Finland: first-hospitalized patients followed up to 24 years. Acta Psychiatr Scand. 2019;139(2):154-163. doi:10.1111/acps.12990 30480317

[4] Nordentoft M, Mortensen PB, Pedersen CB. Absolute risk of suicide after first hospital contact in mental disorder. Arch Gen Psychiatry. 2011;68(10):1058-1064. doi:10.1001/archgenpsychiatry.2011.113 21969462

[5] Madsen T, Erlangsen A, Hjorthøj C, Nordentoft M. High suicide rates during psychiatric inpatient stay and shortly after discharge. Acta Psychiatr Scand. 2020;142(5):355-365. doi:10.1111/acps.13221 32715465

[6] Pirkola S, Sohlman B, Heilä H, Wahlbeck K. Reductions in postdischarge suicide after deinstitutionalization and decentralization: a nationwide register study in Finland. Psychiatr Serv. 2007;58(2):221-226. doi:10.1176/ps.2007.58.2.221 17287379

[7] Chung D, Hadzi-Pavlovic D, Wang M, Swaraj S, Olfson M, Large M. Meta-analysis of suicide rates in the first week and the first month after psychiatric hospitalisation. BMJ Open. 2019;9(3):e023883. doi:10.1136/bmjopen-2018-023883 30904843 PMC6475206

[8] Large M, Sharma S, Cannon E, Ryan C, Nielssen O. Risk factors for suicide within a year of discharge from psychiatric hospital: a systematic meta-analysis. Aust N Z J Psychiatry. 2011;45(8):619-628. doi:10.3109/00048674.2011.590465 21740345

[9] Chung DT, Ryan CJ, Hadzi-Pavlovic D, Singh SP, Stanton C, Large MM. Suicide rates after discharge from psychiatric facilities: a systematic review and meta-analysis. JAMA Psychiatry. 2017;74(7):694-702. doi:10.1001/jamapsychiatry.2017.1044 28564699 PMC5710249

[10] Olfson M, Wall M, Wang S, . Short-term suicide risk after psychiatric hospital discharge. JAMA Psychiatry. 2016;73(11):1119-1126. doi:10.1001/jamapsychiatry.2016.2035 27654151 PMC8259698

[11] Bostwick JM, Pabbati C, Geske JR, McKean AJ. Suicide attempt as a risk factor for completed suicide: even more lethal than we knew. Am J Psychiatry. 2016;173(11):1094-1100. doi:10.1176/appi.ajp.2016.15070854 27523496 PMC5510596

[12] Isometsä ET, Lönnqvist JK. Suicide attempts preceding completed suicide. Br J Psychiatry. 1998;173:531-535. doi:10.1192/bjp.173.6.531 9926085

[13] Isometsä E. Suicidal behaviour in mood disorders—who, when, and why? Can J Psychiatry. 2014;59(3):120-130. doi:10.1177/070674371405900303 24881160 PMC4079239

[14] Isometsä ET. Suicides in mood disorders in psychiatric settings in Nordic national register-based studies. Front Psychiatry. 2020;11(721):721. doi:10.3389/fpsyt.2020.00721 32848909 PMC7390882

[15] Franklin JC, Ribeiro JD, Fox KR, . Risk factors for suicidal thoughts and behaviors: a meta-analysis of 50 years of research. Psychol Bull. 2017;143(2):187-232. doi:10.1037/bul0000084 27841450

[16] Hawton K, Casañas I Comabella C, Haw C, Saunders K. Risk factors for suicide in individuals with depression: a systematic review. J Affect Disord. 2013;147(1-3):17-28. doi:10.1016/j.jad.2013.01.004 23411024

[17] Turecki G, Brent DA. Suicide and suicidal behaviour. Lancet. 2016;387(10024):1227-1239. doi:10.1016/S0140-6736(15)00234-2 26385066 PMC5319859

[18] van Heeringen K, Mann JJ. The neurobiology of suicide. Lancet Psychiatry. 2014;1(1):63-72. doi:10.1016/S2215-0366(14)70220-2 26360403

[19] Aaltonen K, Näätänen P, Heikkinen M, . Differences and similarities of risk factors for suicidal ideation and attempts among patients with depressive or bipolar disorders. J Affect Disord. 2016;193:318-330. doi:10.1016/j.jad.2015.12.033 26774520

[20] Melhem NM, Porta G, Oquendo MA, . Severity and variability of depression symptoms predicting suicide attempt in high-risk individuals. JAMA Psychiatry. 2019;76(6):603-613. doi:10.1001/jamapsychiatry.2018.4513 30810713 PMC6551844

[21] Cai Z, Junus A, Chang Q, Yip PSF. The lethality of suicide methods: a systematic review and meta-analysis. J Affect Disord. 2022;300:121-129. doi:10.1016/j.jad.2021.12.054 34953923

[22] Fawcett J, Scheftner WA, Fogg L, . Time-related predictors of suicide in major affective disorder. Am J Psychiatry. 1990;147(9):1189-1194. doi:10.1176/ajp.147.9.1189 2104515

[23] Kleiman EM, Turner BJ, Fedor S, Beale EE, Huffman JC, Nock MK. Examination of real-time fluctuations in suicidal ideation and its risk factors: results from two ecological momentary assessment studies. J Abnorm Psychol. 2017;126(6):726-738. doi:10.1037/abn0000273 28481571

[24] Sund R. Quality of the Finnish Hospital Discharge Register: a systematic review. Scand J Public Health. 2012;40(6):505-515. doi:10.1177/1403494812456637 22899561

[25] Lunetta P, Lounamaa A, Sihvonen S. Surveillance of injury-related deaths: medicolegal autopsy rates and trends in Finland. Inj Prev. 2007;13(4):282-284. doi:10.1136/ip.2006.012922 17686941 PMC2598324

[26] Fauvernier M, Remontet L, Uhry Z, Bossard N, Roche L. survPen: an R package for hazard and excess hazard modelling with multidimensional penalized splines. J Open Source Softw. 2019;4(40). doi:10.21105/joss.01434

[27] Official Statistics of Finland (OSF). Causes of death 2017. Accessed May 29, 2023. https://www.stat.fi/til/ksyyt/2017/ksyyt_2017_2018-12-17_kat_006_fi.html

[28] Aaltonen KI, Isometsä E, Sund R, Pirkola S. Decline in suicide mortality after psychiatric hospitalization for depression in Finland between 1991 and 2014. World Psychiatry. 2018;17(1):110-112. doi:10.1002/wps.20501 29352527 PMC5775129

[29] Isometsä ET, Heikkinen ME, Marttunen MJ, Henriksson MM, Aro HM, Lönnqvist JK. The last appointment before suicide: is suicide intent communicated? Am J Psychiatry. 1995;152(6):919-922. doi:10.1176/ajp.152.6.919 7755124

[30] Deisenhammer EA, Behrndt EM, Kemmler G, Haring C, Miller C. Suicide risk factors in patients recently discharged from a psychiatric hospital: a case-control study. J Clin Psychiatry. 2019;80(5):18m12702. doi:10.4088/JCP.18m12702 31536688

[31] Bolton JM, Gunnell D, Turecki G. Suicide risk assessment and intervention in people with mental illness. BMJ. 2015;351:h4978. doi:10.1136/bmj.h4978 26552947

[32] Gournellis R, Tournikioti K, Touloumi G, . Psychotic (delusional) depression and completed suicide: a systematic review and meta-analysis. Ann Gen Psychiatry. 2018;17:39. doi:10.1186/s12991-018-0207-1 30258483 PMC6150953

[33] Leadholm AK, Rothschild AJ, Nielsen J, Bech P, Ostergaard SD. Risk factors for suicide among 34,671 patients with psychotic and non-psychotic severe depression. J Affect Disord. 2014;156:119-125. doi:10.1016/j.jad.2013.12.003 24388683

[34] Suominen K, Haukka J, Valtonen HM, Lönnqvist J. Outcome of patients with major depressive disorder after serious suicide attempt. J Clin Psychiatry. 2009;70(10):1372-1378. doi:10.4088/JCP.09m05110blu 19906342

[35] Paljärvi T, Tiihonen J, Lähteenvuo M, Tanskanen A, Fazel S, Taipale H. Psychotic depression and deaths due to suicide. J Affect Disord. 2023;321:28-32. doi:10.1016/j.jad.2022.10.035 36280195

[36] Olfson M, Wall M, Wang S, Crystal S, Gerhard T, Blanco C. Suicide following deliberate self-harm. Am J Psychiatry. 2017;174(8):765-774. doi:10.1176/appi.ajp.2017.16111288 28320225

[37] Kessler RC, Bauer MS, Bishop TM, . Evaluation of a model to target high-risk psychiatric inpatients for an intensive postdischarge suicide prevention intervention. JAMA Psychiatry. 2023;80(3):230-240. doi:10.1001/jamapsychiatry.2022.4634 36652267 PMC9857842

[38] Agerbo E. High income, employment, postgraduate education, and marriage: a suicidal cocktail among psychiatric patients. Arch Gen Psychiatry. 2007;64(12):1377-1384. doi:10.1001/archpsyc.64.12.1377 18056545

[39] Agerbo E, Mortensen PB, Eriksson T, Qin P, Westergaard-Nielsen N. Risk of suicide in relation to income level in people admitted to hospital with mental illness: nested case-control study. BMJ. 2001;322(7282):334-335. doi:10.1136/bmj.322.7282.334 11159656 PMC26575

[40] Aaltonen KI, Rosenström T, Jylhä P, . Do suicide attempts of mood disorder patients directly increase the risk for a reattempt? Front Psychiatry. 2020;11:547791. doi:10.3389/fpsyt.2020.547791 33324247 PMC7725715

[41] Olfson M, Cosgrove CM, Altekruse SF, Wall MM, Blanco C. Living alone and suicide risk in the United States, 2008–2019. Am J Public Health. 2022;112(12):1774-1782. doi:10.2105/AJPH.2022.307080 36383944 PMC9670225

[42] Lorant V, Kunst AE, Huisman M, Costa G, Mackenbach J; EU Working Group on Socio-Economic Inequalities in Health. Socio-economic inequalities in suicide: a European comparative study. Br J Psychiatry. 2005;187:49-54. doi:10.1192/bjp.187.1.49 15994571

[43] Qin P, Agerbo E, Mortensen PB. Suicide risk in relation to socioeconomic, demographic, psychiatric, and familial factors: a national register-based study of all suicides in Denmark, 1981-1997. Am J Psychiatry. 2003;160(4):765-772. doi:10.1176/appi.ajp.160.4.765 12668367

