# Supplementary material for: Variations in Suicide Risk and Risk Factors After Hospitalization for Depression in Finland, 1996-2017
Source: JAMA Psychiatry. 2024 Feb 14;81(5):506–15. doi: 10.1001/jamapsychiatry.2023.5512 (PMC10867776; doi:10.1001/jamapsychiatry.2023.5512)
Supplement: Supplement 1. — eTable. Unadjusted and Adjusted Incidence Rate Ratios for Suicide by Cumulative Time Since Discharge eFigure 1. Hazard of Suicide by Age and Days Since Discharge Up to 2 Years eFigure 2. Hazard of Suicide by Days Since Discharge By 11 Factors Up to 2 Years [file jamapsychiatry-e235512-s001.pdf]

Supplemental Online Content

Aaltonen K, Sund R, Hakulinen C, Pirkola S, Isometsä E. Variations in suicide risk and risk factors after hospitalization for depression in Finland, 1996-2017. *JAMA Psychiatry*. Published online February 14, 2024. doi:10.1001/jamapsychiatry.2023.5512

eTable. Unadjusted and Adjusted Incidence Rate Ratios for Suicide by Cumulative Time Since Discharge

eFigure 1. Hazard of Suicide by Age and Days Since Discharge Up to 2 Years

eFigure 2. Hazard of Suicide by Days Since Discharge By 11 Factors Up to 2 Years

This supplementary material has been provided by the authors to give readers additional information about their work.

© 2024 Aaltonen K et al. *JAMA Psychiatry*.

eTable. Unadjusted and Adjusted[a] Incidence Rate Ratios for Suicide by Cumulative Time Since Discharge

	≤ 3 days		≤ 7 days		≤ 30 days		≤ 90 days		≤ 365 days		≤ 2 years	
	IRR (95%CI)	aIRR (95%CI)	IRR (95%CI)	aIRR (95%CI)	IRR (95%CI)	aIRR (95%CI)	IRR (95%CI)	aIRR (95%CI)	IRR (95%CI)	aIRR (95%CI)	IRR (95%CI)	aIRR (95%CI)
Men	1.96 (1.31-2.96)	2.23 (1.48-3.39)	2.07 (1.53-2.80)	2.25 (1.65-3.07)	2.16 (1.79-2.62)	2.33 (1.92-2.84)	2.15 (1.88-2.48)	2.27 (1.97-2.62)	2.21 (1.99-2.45)	2.30 (2.07-2.57)	2.20 (2.01-2.40)	2.23 (2.03-2.45)
Depression severity[b]												
Severe	2.18 (1.37-3.52)	1.92 (1.19-3.15)	1.69 (1.21-2.36)	1.60 (1.13-2.28)	1.53 (1.25-1.88)	1.56 (1.26-1.93)	1.30 (1.11-1.51)	1.36 (1.16-1.59)	1.29 (1.16-1.45)	1.40 (1.25-1.58)	1.24 (1.12-1.37)	1.36 (1.22-1.50)
Psychotic	2.36 (1.35-4.07)	1.88 (1.03-3.40)	1.73 (1.14-2.57)	1.54 (0.98-2.38)	1.37 (1.05-1.78)	1.29 (0.96-1.71)	1.33 (1.09-1.60)	1.37 (1.11-1.69)	1.37 (1.19-1.58)	1.45 (1.24-1.69)	1.30 (1.15-1.47)	1.41 (1.23-1.61)
AUD	0.43 (0.18-0.86)	0.32 (0.13-0.65)	0.94 (0.61-1.40)	0.70 (0.44-1.06)	1.04 (0.80-1.33)	0.75 (0.57-0.98)	1.47 (1.23-1.76)	0.92 (0.77-1.11)	1.43 (1.26-1.63)	0.99 (0.87-1.13)	1.39 (1.24-1.55)	1.02 (0.91-1.15)
SUD[c]	0.93 (0.29-2.23)	1.16 (0.35-2.84)	0.89 (0.38-1.75)	0.96 (0.40-1.91)	1.35 (0.89-1.95)	1.37 (0.90-2.01)	2.52 (1.68-3.67)	1.22 (0.90-1.63)	2.22 (1.63-2.96)	1.47 (1.19-1.80)	2.05 (1.56-2.65)	1.59 (1.33-1.88)
GAS												
20-49	2.85 (1.59-5.66)	2.94 (1.61-5.92)	2.12 (1.42-3.29)	2.10 (1.39-3.29)	1.57 (1.24-2.01)	1.54 (1.21-1.99)	1.47 (1.24-1.76)	1.42 (1.19-1.70)	1.44 (1.26-1.64)	1.33 (1.16-1.52)	1.39 (1.24-1.55)	1.29 (1.15-1.45)
0-19	5.26 (1.46-15.39)	4.19 (1.11-12.94)	2.89 (1.55-8.48)	3.15 (1.22-7.14)	3.56 (2.15-5.64)	2.96 (1.75-4.79)	1.88 (1.41-2.48)	2.01 (1.32-2.98)	1.66 (1.33-2.06)	1.61 (1.17-2.17)	2.05 (1.56-2.65)	1.51 (1.14-1.98)

© 2024 Aaltonen K et al. *JAMA Psychiatry*.

	≤ 3 days		≤ 7 days		≤ 30 days		≤ 90 days		≤ 365 days		≤ 2 years	
	IRR (95%CI)	aIRR (95%CI)	IRR (95%CI)	aIRR (95%CI)	IRR (95%CI)	aIRR (95%CI)	IRR (95%CI)	aIRR (95%CI)	IRR (95%CI)	aIRR (95%CI)	IRR (95%CI)	aIRR (95%CI)
Suicide attempt[d]												
At index episode[e]	3.66 (2.09-6.02)	NA	3.08 (2.00-4.56)	NA	2.62 (1.98-3.41)	NA	2.74 (2.24-3.31)	NA	2.77 (2.40-3.19)	NA	2.52 (2.21-2.86)	NA
Hanging/firearms	18.86 (3.10-59.75)	13.70 (2.23-44.59)	10.06 (1.66-31.48)	6.98 (1.15-22.20)	3.85 (0.64-11.96)	2.54 (0.42-7.94)	2.08 (0.35-6.45)	1.42 (0.23-4.40)	3.41 (1.35-6.93)	2.26 (0.90-4.61)	3.55 (1.62-6.62)	2.34 (1.07-4.37)
Posion/Cutting	3.07 (1.63-5.32)	2.67 (1.39-4.75)	2.88 (1.81-4.37)	2.41 (1.49-3.73)	2.63 (1.97-3.46)	2.18 (1.61-2.90)	2.76 (2.24-3.36)	2.30 (1.85-2.82)	2.76 (2.37-3.20)	2.30 (1.97-2.68)	2.52 (2.20-2.88)	2.10 (1.83-2.41)
Other	6.59 (1.08-20.87)	5.71 (0.93-18.33)	3.50 (0.58-10.96)	2.93 (0.48-9.22)	1.98 (0.49-5.17)	1.64 (0.41-4.28)	2.75 (1.25-5.14)	2.30 (1.05-4.31)	2.68 (1.54-4.29)	2.20 (1.26-3.52)	2.25 (1.34-3.51)	1.86 (1.11-2.90)
In past four years[e]	2.09 (1.28-3.29)	2.31 (1.37-3.73)	2.09 (1.46-2.93)	2.26 (1.55-3.24)	2.08 (1.66-2.58)	2.17 (1.71-2.73)	2.20 (1.87-2.58)	2.20 (1.85-2.60)	2.25 (1.99-2.53)	2.16 (1.90-2.46)	2.24 (2.01-2.49)	2.14 (1.91-2.40)
Involuntary admission	1.48 (0.81-2.53)	1.11 (0.59-1.96)	1.36 (0.87-2.06)	1.08 (0.67-1.67)	1.46 (1.12-1.88)	1.21 (0.91-1.59)	1.36 (1.11-1.64)	1.11 (0.90-1.36)	1.53 (1.33-1.75)	1.25 (1.08-1.44)	1.54 (1.37-1.73)	1.29 (1.14-1.45)
Age[f]												
Middle age (40-64)	2.76 (1.68-4.75)	2.80 (1.67-4.88)	2.05 (1.45-2.95)	2.04 (1.43-2.97)	1.70 (1.38-2.11)	1.75 (1.41-2.19)	1.48 (1.28-1.73)	1.50 (1.28-1.75)	1.35 (1.21-1.51)	1.38 (1.23-1.55)	1.36 (1.23-1.50)	1.40 (1.26-1.54)
Advanced age ((≥65)	2.45 (1.28-4.68)	3.34 (1.68-6.64)	1.76 (1.09-2.78)	2.52 (1.53-4.11)	1.39 (1.04-1.86)	2.02 (1.47-2.74)	1.18 (0.94-1.46)	1.70 (1.34-2.14)	1.12 (0.95-1.31)	1.59 (1.33-1.89)	1.03 (0.89-1.20)	1.47 (1.26-1.72)
Living alone	0.80 (0.52-1.20)	0.87 (0.56-1.33)	0.72 (0.52-0.98)	0.71 (0.51-0.97)	0.78 (0.65-0.95)	0.72 (0.59-0.88)	0.89 (0.77-1.02)	0.81 (0.70-0.94)	1.04 (0.94-1.16)	0.93 (0.84-1.04)	1.12 (1.02-1.22)	1.00 (0.91-1.09)

© 2024 Aaltonen K et al. *JAMA Psychiatry*.

	≤ 3 days		≤ 7 days		≤ 30 days		≤ 90 days		≤ 365 days		≤ 2 years	
	IRR (95%CI)	aIRR (95%CI)	IRR (95%CI)	aIRR (95%CI)	IRR (95%CI)	aIRR (95%CI)	IRR (95%CI)	aIRR (95%CI)	IRR (95%CI)	aIRR (95%CI)	IRR (95%CI)	aIRR (95%CI)
Household's disposable income level[g]												
Middle tertile	1.08 (0.69-1.73)	0.83 (0.52-1.34)	0.78 (0.57-1.09)	0.61 (0.44-0.86)	0.72 (0.59-0.87)	0.58 (0.47-0.71)	0.70 (0.61-0.81)	0.60 (0.51-0.69)	0.65 (0.59-0.73)	0.58 (0.52-0.65)	0.67 (0.62-0.74)	0.62 (0.56-0.68)
Highest tertile	1.99 (1.04-3.67)	1.25 (0.62-2.46)	1.40 (0.86-2.20)	0.86 (0.50-1.41)	0.92 (0.65-1.26)	0.56 (0.39-0.80)	0.91 (0.71-1.15)	0.61 (0.47-0.79)	0.71 (0.59-0.86)	0.52 (0.42-0.63)	0.71 (0.60-0.83)	0.55 (0.46-0.66)
Education level[h]												
Upper secondary	1.04 (0.65-1.65)	1.17 (0.73-1.90)	1.12 (0.80-1.57)	1.26 (0.89-1.80)	1.05 (0.85-1.30)	1.17 (0.94-1.46)	1.10 (0.94-1.28)	1.21 (1.03-1.42)	1.05 (0.94-1.18)	1.17 (1.04-1.32)	1.10 (1.00-1.22)	1.21 (1.09-1.34)
Tertiary[i]	1.40 (0.81-2.36)	1.30 (0.73-2.28)	1.45 (0.97-2.14)	1.54 (1.00-2.34)	1.42 (1.11-1.81)	1.70 (1.31-2.21)	1.42 (1.18-1.70)	1.71 (1.40-2.07)	1.30 (1.14-1.49)	1.68 (1.45-1.94)	1.23 (1.09-1.39)	1.56 (1.37-1.77)
Duration of hospitalization[j]												
1-3 weeks	0.86 (0.52-1.43)	0.68 (0.41-1.14)	1.19 (0.82-1.73)	1.01 (0.69-1.48)	1.07 (0.85-1.35)	0.96 (0.76-1.22)	0.98 (0.83-1.16)	0.91 (0.77-1.08)	0.90 (0.79-1.03)	0.86 (0.75-0.98)	0.90 (0.81-1.01)	0.87 (0.78-0.98)
>3 weeks	0.96 (0.59-1.55)	0.56 (0.33-0.95)	1.04 (0.72-1.52)	0.74 (0.49-1.10)	0.95 (0.75-1.20)	0.76 (0.59-0.97)	0.89 (0.75-1.06)	0.77 (0.64-0.92)	0.91 (0.81-1.04)	0.81 (0.71-0.93)	0.90 (0.80-1.00)	0.82 (0.73-0.93)
Year of hospitalization[k]												
2003-2009	0.62 (0.38-0.99)	0.58 (0.36-0.93)	0.68 (0.48-0.96)	0.63 (0.44-0.90)	0.69 (0.55-0.86)	0.63 (0.51-0.79)	0.76 (0.65-0.89)	0.70 (0.60-0.83)	0.79 (0.70-0.89)	0.72 (0.64-0.81)	0.80 (0.72-0.89)	0.74 (0.66-0.82)
2010-2017	0.56 (0.33-0.91)	0.46 (0.27-0.76)	0.59 (0.41-0.85)	0.51 (0.35-0.74)	0.63 (0.50-0.79)	0.54 (0.43-0.69)	0.67 (0.56-0.79)	0.58 (0.48-0.69)	0.66 (0.58-0.75)	0.57 (0.50-0.65)	0.67 (0.60-0.75)	0.58 (0.52-0.65)

© 2024 Aaltonen K et al. *JAMA Psychiatry*.

AUD: alcohol use disorder; aIRR: adjusted incidence rate ratio; CI: confidence interval; GAS: global assessment scale; IRR: incidence rate ratio; NA: Not applicable; SUD: substance use disorder

[a] adjusted for all other variables

[b] all other types of depression (from mild to moderate, partial remissions, other specified, and unspecified) as reference

[c] excluding alcohol or nicotine harmful use or dependence syndrome

[d] categories of suicide attempt at index episode and in past four years non-overlapping, an individual may have both

[e] any method

[f] age 18-39 reference

[g] lowest tertile reference, thresholds correspond to the respective levels of household's disposable income of the entire population

[h] basic education level reference

[i] lowest level tertiary education or higher

[j] ≤1 week reference

[k] years 1996-2002 reference

© 2024 Aaltonen K et al. *JAMA Psychiatry*.

eFigure 1. Hazard of Suicide by Age and Days Since Discharge Up to 2 Years

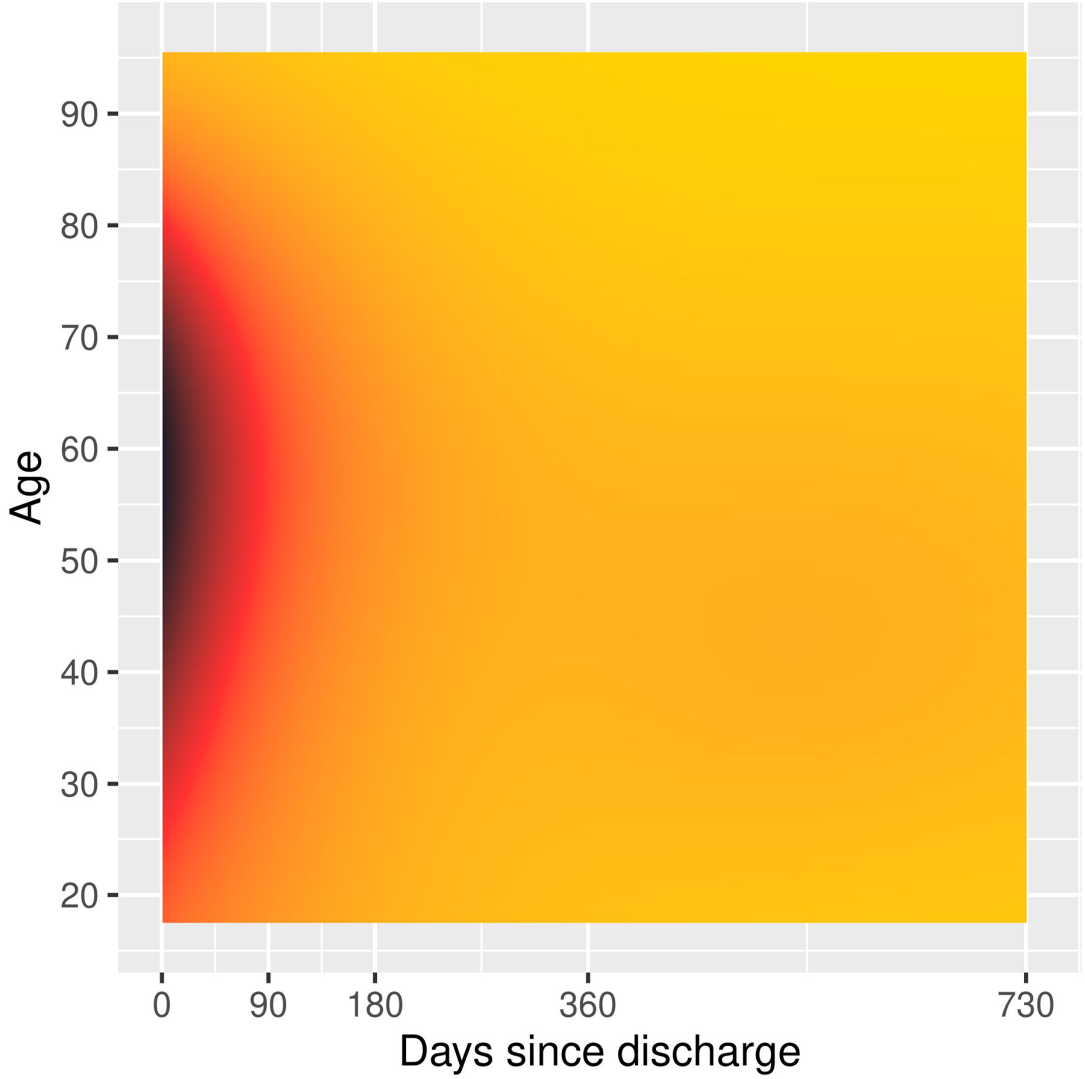

The heat map above represents risk of suicide by age (y-axis) and time from discharge (x-axis), where black indicates higher and yellow lower relative risk.

© 2024 Aaltonen K et al. *JAMA Psychiatry*.

eFigure 2. Hazard of Suicide by Days Since Discharge By 11 Factors Up to 2 Years

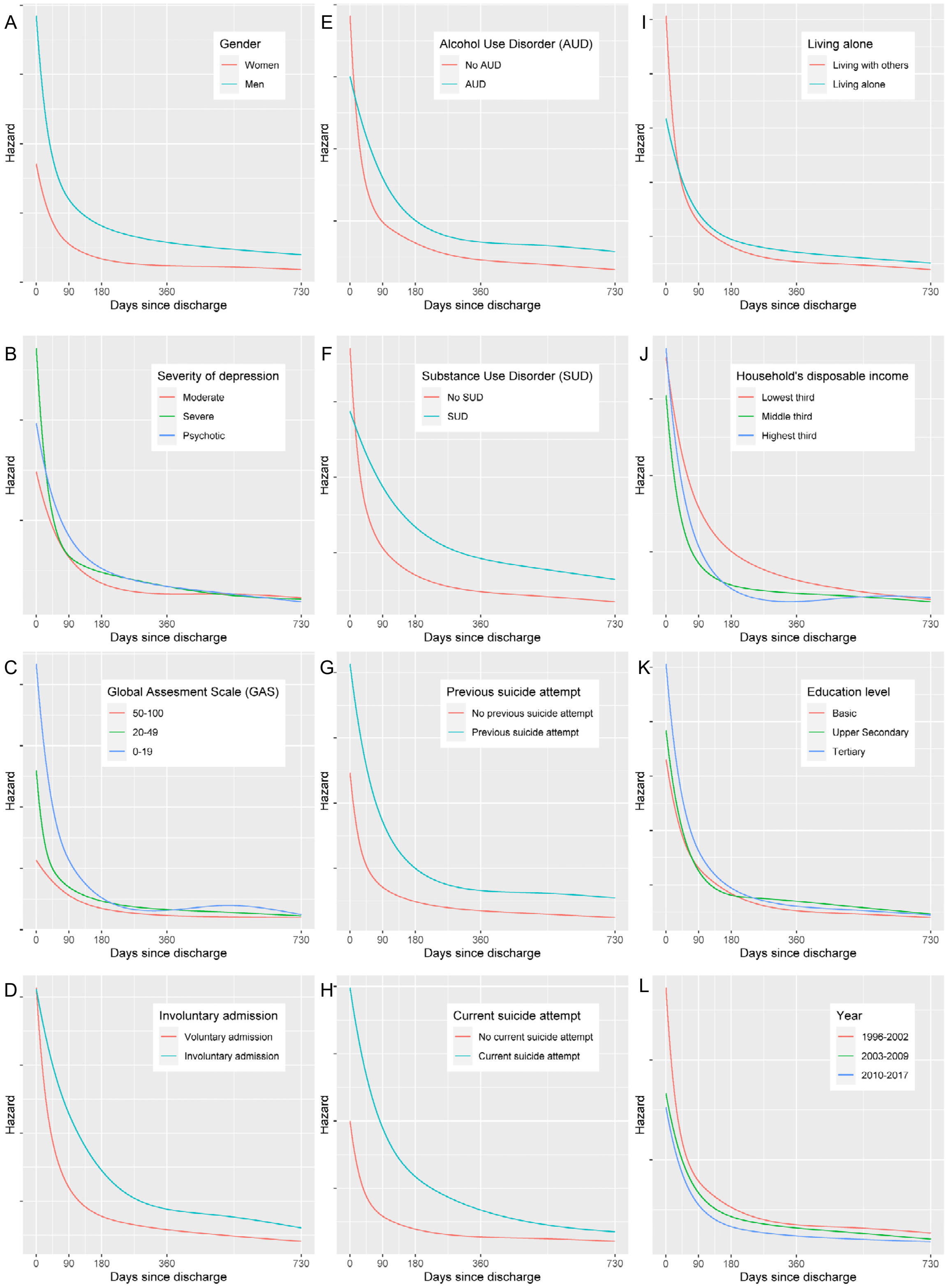

© 2024 Aaltonen K et al. *JAMA Psychiatry*.
