# Supplementary material for: Variations in Suicide Risk and Risk Factors After Hospitalization for Depression in Finland, 1996-2017
Source: JAMA Psychiatry. 2024 Feb 14;81(5):506–15. doi: 10.1001/jamapsychiatry.2023.5512 (PMC10867776; doi:10.1001/jamapsychiatry.2023.5512)
Supplement: Supplement 2. — Data Sharing Statement [file jamapsychiatry-e235512-s002.pdf]

Data Sharing Statement

Aaltonen. Variations in Suicide Risk and Risk Factors After Hospitalization for Depression in Finland, 1996-2017. *JAMA Psychiatry*. Published February 14, 2024.
doi:10.1001/jamapsychiatry.2023.5512

Data
Data available: No

Additional Information
Explanation for why data not available: Availability of data precluded by the Finnish legislation and research permits.
